# Earliest evidence of pollution by heavy metals in archaeological sites

**DOI:** 10.1038/srep14252

**Published:** 2015-09-21

**Authors:** Guadalupe Monge, Francisco J. Jimenez-Espejo, Antonio García-Alix, Francisca Martínez-Ruiz, Nadine Mattielli, Clive Finlayson, Naohiko Ohkouchi, Miguel Cortés Sánchez, Jose María Bermúdez de Castro, Ruth Blasco, Jordi Rosell, José Carrión, Joaquín Rodríguez-Vidal, Geraldine Finlayson

**Affiliations:** 1 Departamento de Cristalografía, Mineralogía y Química Agrícola, Facultad de Química, Universidad de Sevilla, Spain; 2 Department of Biogeochemistry, Japan Agency for Marine-Earth Science and Technology, Yokosuka, Japan; 3 Department of Geographical and Earth Sciences, University of Glasgow, UK; 4 Instituto Andaluz de Ciencias de la Tierra CSIC-UGR, Granada, Spain; 5 Laboratoire G-Time, DSTE, Université Libre Bruxelles (ULB), Brussels, Belgium; 6 The Gibraltar Museum, Gibraltar, UK; 7 Institute of Life and Earth Sciences, The University of Gibraltar, Gibraltar; 8 Departamento de Prehistoria y Arqueología, Facultad de Geografía e Historia, Universidad de Sevilla, Sevilla, Spain; 9 Centro Nacional de Investigación sobre la Evolución Humana (CENIEH) Burgos, Spain; 10 University College London Anthropology, London, UK; 11 Departament de Prehistòria, Universitat Autònoma de Barcelona, Barcelona, Spain; 12 Àrea de Prehistòria, Universitat Rovira i Virgili (URV), Tarragona, Spain; 13 Institut Català de Paleoecologia Humana i Evolució Social (IPHES), Tarragona, Spain; 14 Departamento de Biología Vegetal, Universidad de Murcia, Murcia, Spain; 15 Departamento de Geodinámica y Paleontología, Facultad de Ciencias Experimentales, Universidad de Huelva, Huelva, Spain

## Abstract

*Homo* species were exposed to a new biogeochemical environment when they began to occupy caves. Here we report the first evidence of palaeopollution through geochemical analyses of heavy metals in four renowned archaeological caves of the Iberian Peninsula spanning the last million years of human evolution. Heavy metal contents reached high values due to natural (guano deposition) and anthropogenic factors (e.g. combustion) in restricted cave environments. The earliest anthropogenic pollution evidence is related to Neanderthal hearths from Gorham's Cave (Gibraltar), being one of the first milestones in the so-called “Anthropocene”. According to its heavy metal concentration, these sediments meet the present-day standards of “contaminated soil”. Together with the former, the Gibraltar Vanguard Cave, shows Zn and Cu pollution ubiquitous across highly anthropic levels pointing to these elements as potential proxies for human activities. Pb concentrations in Magdalenian and Bronze age levels at El Pirulejo site can be similarly interpreted. Despite these high pollution levels, the contaminated soils might not have posed a major threat to *Homo* populations. Altogether, the data presented here indicate a long-term exposure of *Homo* to these elements, via fires, fumes and their ashes, which could have played certain role in environmental-pollution tolerance, a hitherto neglected influence.

The expansion of human industrial activity, including mining, smelting, and synthetic compound creation, has caused an exponential increase in the amounts of heavy metals released to the atmosphere, water, and soil^1^. This increase is a major threat for human health.

Although the adaptive capabilities of our species at the multi-millennial scale are far from understood, it is clear that human communities have been in close contact with heavy metals ever since the origin of mining explotation during the Chalcolithic, and evidence abounds in southwestern Iberia from c. 5000 years BP^2^. That kind of pollution was triggered in western Europe around 4000 years BP or slightly before^3^. However, the subject has not hitherto been considered through specifically orientated investigations from Neolithic backwards^2^,^3^, and this is the main goal here. Therefore, assessing the concentration of heavy metals in caves and rockshelters with archaeological and palaeoanthropological evidence is a pertinent matter of study. Caves are persistently restricted environment and heavy metal bio-mediated accumulations, such as those caused by natural organic sources (bird and bat guano)^4^,^5^, or by inorganic sources pertinent to the geological context of the cavern^6^,^7^, may reach relatively high levels. Increases of these pollutants can be expected from long-term human activities, especially combustion, which has been reported in connection with ash biomass (heavy metal enrichment mainly dealing with Cu, Zn and Mn)^8^ and demonstrated to show variable toxicity.

In the past, the use of fire might have provided major adaptive advantages to humans^9^ and may have promoted sociability^10^. However, whenever *Homo* species may have begun to use open fires in the restricted environments of caves, it is clear they became to some extent exposed to pollutants. Biomass cooking, using open fires or rudimentary stoves, is still a common practice in certain societies. Apart from unstudied, unpredictable, long-term effects, pollution from combustion can be associated with up to 1.9 million premature deaths every year, as well as chronic and acute respiratory illnesses, and it is the 4^th^ major cause of morbidity globally^11^. Indeed, the decrease of open fire exposure has been regarded equivalent to smoking cessation^12^.

This paper is aimed at evaluating the occurrence of high heavy metal levels in archaeological sites inhabited by *Homo* (Fig. 1). In order to reach this objective several cave deposits have been geochemically analysed, including representative archaeological sites from the Iberian Peninsula persistently occupied by *Homo* populations during the last 1.4 Ma^13^,^14^,^15^. In addition, experimental fires were performed in order to test the relationships between wood ashes and high heavy metal concentrations in cave sediments. Zn isotopic analysis have been carried out in order to evaluate whether element accumulation is derived from sea spray or affected by diagenetic processes.

## Results

Gran Dolina shows high EF values (Fig. 2 and Supplementary Table S1) for Cu and Zn: EF_Zn_ = 9.2 at level TD 9 and EF_Zn_ = 4.5 at level TD 6–2, and EF_Cu_ = 5.8–2.8 at level TD 9. Ni values are noteworthy only for two samples. Values higher than those from the established geochemical baseline (Supplementary Table S1) suggest that Gran Dolina cave sediments are mainly enriched in Ni, Zn, and Cu compared with other external deposits.

Gorham’s Cave shows the highest EF_Zn_, EF_Cu_, and EF_Ni_ from all sites (Fig. 2 and Supplementary Table S1). The samples GOR-2 and GOR-1 deserve special mention, showing EF_Zn_ = 74.3, EF_Cu_ = 66.4 and EF_Ni_ = 17.0 at Level IV for GOR-2, while GOR-1 exhibits EF_Zn_ = 20.7, EF_Cu_ = 5.6 and EF_Ni_ = 2.6 at the same level. The remaining samples show high to moderate values of Cu and Zn, and occasionally moderate Ni concentrations. These pollutants depict a strong relationship to each other, suggesting a common source. Heavy metal concentrations in some cases exceeded the values of the Soil Clean up Criteria from the New Jersey Department of Environmental Protection (SCC NJDEP)^16^ (Zn* > 1500 ppm; Cu* > 600 ppm; Ni > 250 ppm; *Criterion based on ecological effects), and pointed towards potential polluted levels, and the subsequent health risk for living organisms.

At Vanguard Cave, samples 9 and 10 are worth mentioning because of their high pollutant values at level 9 (Fig. 3 and Supplementary Table S1), mainly on EF_Zn_ and EF_Cu_. EF_Zn_ values are 4.1 and 3.7 from level 9 while the highest EF_Cu_ value is 5.0 at the same level. The remaining levels show moderate pollutant concentrations, mainly composed by Cr and Ni, and occasionally Zn.

The main pollutants at El Pirulejo are Pb and Ni, followed by Cr (Fig. 2 and Supplementary Table S1). EF_Pb_ = 3.6 at level I, and EF_Pb_ = 3.3–3.2 at levels I and III respectively, show the highest Pb values. These samples are from the uppermost part of the deposit, while Pb fails to be recorded 200 cm depth downwards (level IV). With respect to Ni, the highest values are reported at level IV with EF_Ni_ = 4.3 and level III with EF_Ni_ = 3.3. Unlike Pb, Ni occurs at the bottom, where it becomes the only pollutant. Relatively high values of Cr (EF) are only present in two samples of the top part of the profile, namely EF_Cr_ = 2.4 at level III, and EF_Cr_ = 2 at level IV.

## Discussion

The studied sites fail to develop soil *sensu stricto* because endokarstic deposits undergo different sedimentary processes from those affecting conventional soils. Although they can experience either post-depositional geogenic or pedogenic effects, it is noteworthy that these environments are less subaerially exposed and less affected by atmospheric weathering processes (freeze-thaw, solifluction, leaching and cementation). Therefore, endokarstic deposits develop less pedological postdepositional modifications than open air sites^17^.

**Early-Middle Palaeolithic.** Gran Dolina site recorded industries starting from the Lower Pleistocene, from about 1.0 to 0.125 Ma^18^. High EF values of Zn and Cu were founded at level TD 9 (Fig. 2 and Supplementary Table S1). This level TD 9 is located near to a guano deposit with an age of 0.45 Ma^19^,^20^. These data are in agreement with previous endokarstic geochemical studies containing bat guano, showing Cu and Zn pollution in the vicinity of the dung^5^. Although modern deposits of bat guano are enriched in N and P as primary geochemical signal, deposits of diagenetically altered guano are enriched in Zn and Cu due to increased organic matter degradation associated with decreased availability of nitrogen and sulphur^21^. It ought to be highlighted that the time estimation for this early stages of diagenesis associated with organic matter degradation is only of decades. This implies that the geochemical values that persist in the archaeological record do indeed record the palaeochemistry of the sediment near the time of its deposition^21^.

Zn and Cu are essential micronutrients for plant development and activation of enzymes; it has also been documented that the concentration of these metals or elements in plants is closely related to the levels of the elements in the soil^22^. In the studied site, only the closed samples TD9-10 and TD9-11 show an increase in metals plausibly related to guano (Fig. 2 and Supplementary Table S1). The Pearson coefficient (Cu-Zn = 0.95) confirms the common distribution of these heavy metals in Gran Dolina and points that the Cu and Zn signal due to guano has been preserved and did not suffer differential leaching. Thus, the overall pattern of the pollutant behavior observed for Gran Dolina suggests low mobility of heavy metals in the profile, associated with the occurrence of carbonates and organic matter.

From an archaeological point of view, the bat guano deposits do not point towards an occasional compost of the cave by the inhabitants, because the accumulation of appreciable amounts of guano is only possible during non-occupational periods^23^. In fact, the level TD-9 has not yielded any fossil remains^24^. Later on, *Homo* populations occupied the cave, and three sublevels inside TD-10 with abundant fossil remains and lithic industry can be identified^25^. Nevertheless, human inhabitants were not in contact with the studied palaeoguano deposit because it was sealed. In any case, this study confirms that guano deposits, ubiquitous in caves, represent the source for the first heavy metal input to *Homo* environments.

**Middle Palaeolithic.** Deposits have been studied at Gorham’s (level IV) and Vanguard Caves, where well-preserved hearths have been reported^26^. Sediment samples with outstanding heavy metal contents (V-9; V-10 and V-21 from Vanguard and GOR-1 and GOR-2 from Gorham’s) have been collected at the levels precisely characterized by occurrence of these hearths (Figs. 2 and 3). In the case of a hearth sample from Gorham’s Cave (GOR-2) the values reached (Ni = 493.8 ppm; Cu = 1592.6 ppm; Zn = 4158.1 ppm) can be considered as a Cu-Zn-Ni polluted soil by modern criteria^16^, and it becomes the oldest documented evidence of pollution generated by *Homo*, and perhaps a milestone in the so-called Anthropocene^27^.

The values obtained at Gorham’s hearth are so high that require a detailed explanation. The presence of Zn, Ni and Cu in wood ash remains are related to their presence in plants as micronutrients, but these element accumulations might be also promoted by sea spray, percolation and accumulated by hearths active carbon.

In order to discuss metal sources, Zn isotopic analysis was performed, which is a robust proxy to identify anthropogenic Zn origin^28^. The Zn isotopic values were obtained from two samples (with 8 replicates) GOR-2 (spliced as GOR-2a and GOR 2b) and GOR-12 (Supplementary Table S2). Both correspond to samples with high EF_Zn_, and GOR-2 was recovered from a Neanderthal well preserved hearth (Fig. 4). Values from GOR-2 and GOR-12 reached δ^66^Zn_JMC 3-0749_ _L_ = + 0.79 ± 0.02‰ (2 SD) (n = 4) and δ^66^Zn_JMC 3-0749L_ = + 0.52 ± 0.02‰ (2SD) (n = 3), respectively (Supplementary Table S2). Temperatures in open fires do not reach the 906°C required for Zn vaporization and Zn isotope fractionation, but if it occurred it can explain why we reached heavy values in fire residues because light fly ashes show light values^28^. Therefore, hearths preserve an original isotopic signal that allows us to discard a marine source [δ^66^Zn = + 0.3–0.4‰ for the marine soluble fraction of Atlantic marine aerosols^29^] and suggests altered organic matter as main origin, similar to deep soils^30^ [δ^66^Zn = + 0.22 to + 0.76‰].

Organic guano, linked to birds and bats appeared at Level I and II of Gorham’s Cave. No evidence of Zn and Cu percolation from these recent deposits have been found, and pollen and macro-botanical stratigraphy is well established for the Gorham’s Cave levels without any suggestion of particle percolation^31^. Previous micromorphological work indicates extensive diagenesis and the presence of charred and rubefied guano in most combustion zones^32^. Therefore, Gorham’s high levels seem to be related to hearth reutilization, occurrence of fires within palaeoguano substrate and diagenesis.

Another important finding in this site is that the highest Zn and Cu contents are not only found at well-defined hearths, but along entire levels in both caves. This is also exemplified in Vanguard Cave, level 9 (Fig. 3, shaded level), where the highest Zn levels are located and where evidence of human activities (bone remains, tools) has been observed. The absence of these enrichments in levels above and below the hearths points towards an *in situ* enrichment since no migration of these elements can be detected along the sedimentary profile. The pattern of Zn along the entire level can be attributed to different mechanisms like ash fly and/or wood ash redistribution. Wood ash fly is also enriched in Zn and Cu and would be distributed along the cave when the fire was active by convection. The studied caves do not show vertical cracks or chimneys, and fumes must go along the entire cave. Redistribution of ashes from hearths to sleeping areas has been described previously in Neanderthal sites^33^. The use of ash has been linked to their thermal and aseptic properties. This evidence indicates that Zn and Cu sediment content can be used as an anthropic proxy, when extensive diagenesis and/or guano inputs can be discarded.

In this sense, the experimental fires conducted with wood from three different tree species growing in limestones in a National Park (far from potential pollution sources), showed heavy metal enrichment with respect to the geochemical base line (Supplementary Table S1), specially of Cu and Zn. The continuous fires developed in the same cave hearths, as well as the effect of ash spreading in the ground of sleeping areas by *Homo* populations^33^, contributed to the concentration of these elements in the soils, and therefore, the risk of heavy metal exposure increased as well. In those levels with human-activity evidence, the concentration of the heavy metals in hearth levels, as well as in laterally equivalent levels, might depend on the continuity of the hearths through time.

All these data show that Neanderthals were exposed to an environment enriched in heavy metals and fumes inside the caves they occupied. Although in some cases, some of these levels can be considered polluted (according to criteria based on ecological effects^16^), it is not possible to conclude whether they reached toxic levels with only this evidence. However, this environment might have made the Zn exposure worse in the case of Neanderthals as their diet had a high cosumption of shellfish^34^, marine resources^35^, and red meat^36^, related to very high Zn intake. Zn is an important micronutrient and its consumption is vital for human reproduction and long-term evolution^37^; however, high Zn intake can cause chronic Zn toxicity triggering anemia and impaired immune function, but normally Zn excess is excreted (European Union Comission- Scientific Committee on Food)^38^.

It is expected that fumes from fires should be the most damaging factor in these restricted cave environments because ash fly contain higher levels of dioxins and heavy metals than the bottom ash^39^. Neanderthals and anatomical modern humans used fires from at least 300 kyr in a complex way^40^,^41^. It is worth questioning if long-term Neanderthal exposure to fire-derived contaminants might have played an important role in their history. Plausibly, for a limited metapopulation^42^, a decrease in fertility associated to smoke^43^ might have played a role in Neanderthal population dynamics. Interestingly, recent studies have demonstrated that several Neanderthal-derived alleles were affected by smoking behaviour, suggesting that Neanderthal alleles continued to shape human biology^44^.

**Upper Palaeolithic.** Levels represented by El Pirulejo^45^ are quite different to the previous ones (Fig. 2) since guano and well defined hearths are not present. As postulated in a former study^46^, levels of Pb at level P/3 (Upper Magdalenian) are outstanding, which can be linked to the use of galena. A few fragments of this mineral have been recovered in this site. Galena is lead sulphide (PbS) that has been used since prehistoric times as a source of pigment, as a raw material to manufacture beads, pendants or other objects, and to sprinkle over the dead in mortuary ceremonies^47^. The latter was the case of its earliest identified use in El Mirón cave (Spain), associated with an Upper Palaeolithic burial (Magdalenian)^48^. Nevertheless, the presence of Bronze Age burials in upper levels and the concentration of Pb located just at P/3 to P/2 transition could also indicate contamination by degradation and leaching of galena fragments or other metal objects from the upper burials.

In resume, We have documented evidence of palaeopollution in *Homo* home environments, which in some cases were related to *Homo* activities. Data obtained from Gran Dolina indicate that caves contain deposits enriched in heavy metals due to diagenetical processes affecting bat guano, but direct contact with *Homo* did not take place at this site. Middle Palaeolithic (Neanderthal) populations from Gorham’s and Vanguard Caves appear to have lived in environments with high levels of Cu and Zn due to combustion activities, promoted by fly ash and wood ash redistribution, and associated with guano altered deposits. The difference in these element contents between both Gibraltar caves can be related to a higher occupation/re-utilization in Gorham’s and/or higher input from guano deposits (including charred and rubefied guano), not present in Vanguard. The high concentration of Zn-Cu in Gorham’s can have also been promoted by compaction and diagenesis from the original deposits.

This ubiquity of certain heavy metals allows us to identify these elements as an anthropogenic geochemical proxy when the sedimentary input of guano and extensive diagenesis is discarded. The highest Pb content and galena mineral is found during the Upper Palaeolithic at the site of El Pirulejo, suggesting a common use of this mineral.

All these data indicate that *Homo* species inhabited caves with high heavy metal levels, at least from the Middle Palaeolithic. Therefore, the real influence of long-term heavy metal soil exposure may have been very limited, but it depends on how they interacted with these sediments.

## Methods

### Site locations

**Figure 1:**
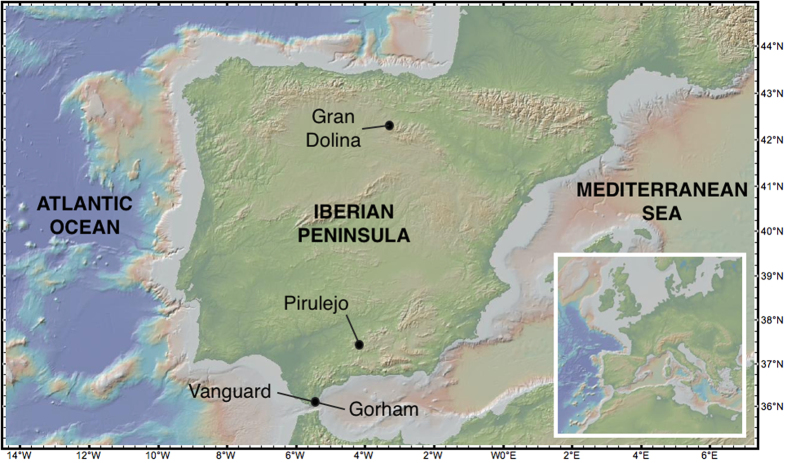
Location of the studied archaeological sites. Map created with GeoMapApp (http://www.geomapapp.org/)^60^.

**Figure 2:**
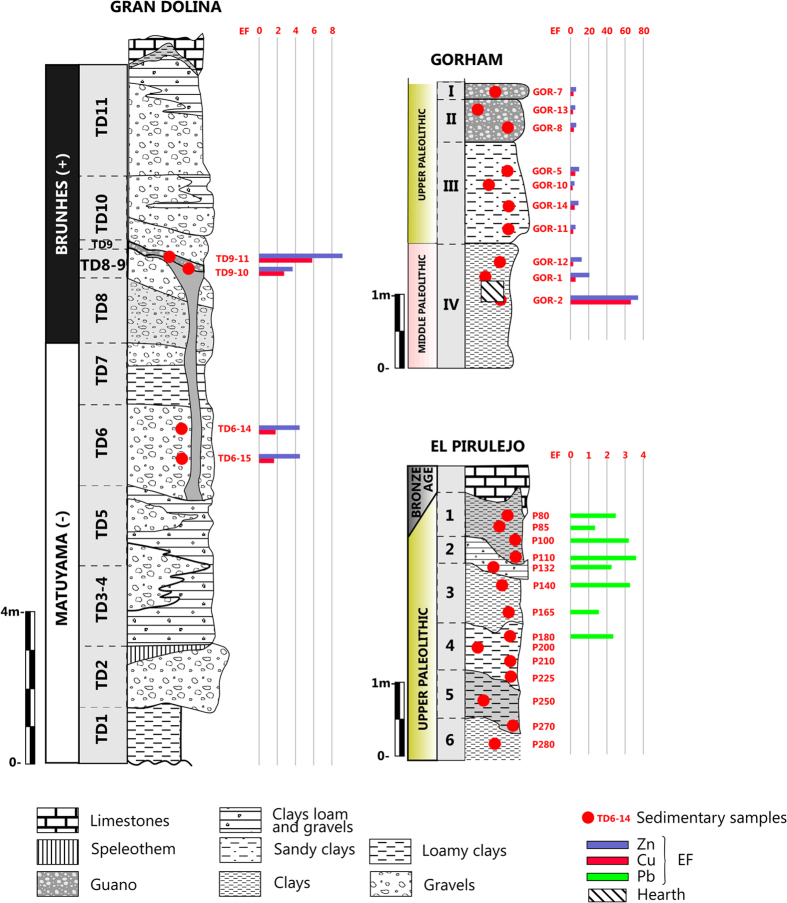
Synthetic stratigraphic columns of Gran Dolina, El Pirulejo and Gorham’s Cave with the situation of the analyzed samples and Enrichment Factor (EF) values.

**Figure 3:**
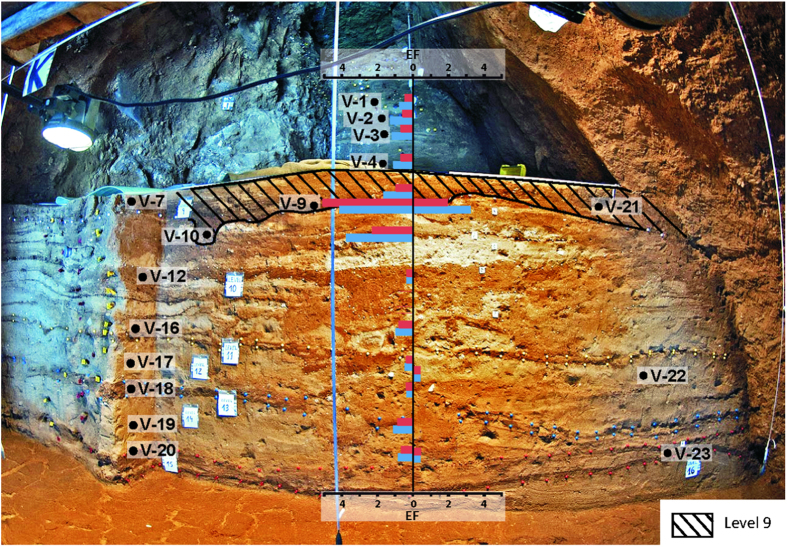
Detailed stratigraphic profile of Vanguard cave with the situation of the analyzed samples and Enrichment Factor (EF) values (Zn values in blue and Cu values in red). We acknowledge Dr. C. Finlayson and archeological team for Vanguard´s stratigraphy picture.

**Figure 4:**
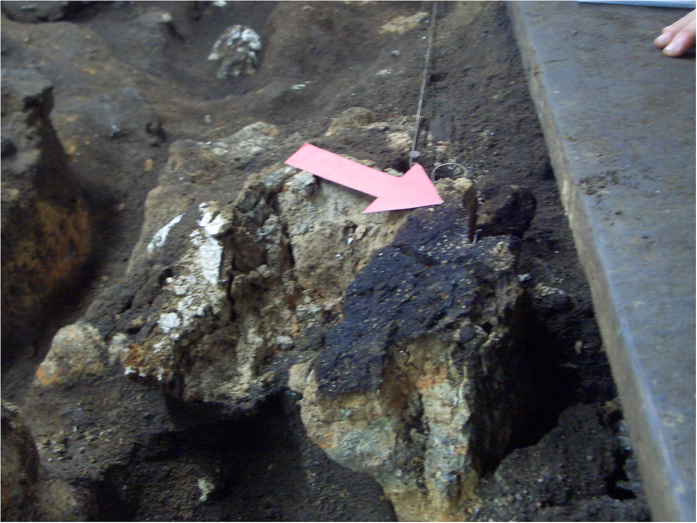
Detailed image of Neanderthal hearth from Gorham’s Cave (Red arrow) where samples GOR-1 and GOR-2 were recovered. We acknowledge Dr. C. Finlayson and archaeological team for Gorham’s picture.

The Gran Dolina site (42° 21’N; 03° 31’W and 980 m asl) (Fig. 1) is located in the Sierra the Atapuerca from northern Spain, about 14 km eastwards Burgos. The region is composed of karstified Cretaceous limestone filled up by Quaternary sedimentary deposits. This well-known palaeoanthropological and archaeological site is composed of eleven Pleistocene stratigraphic levels (dating back from ∼1 Ma to 0.13 Ma) with a thickness of 14 metres. *Homo antecessor* remains were found in the TD-6.2 level^49^,^50^.

Gorham’s Cave (36° 07´N, 05° 20’W and 5 m asl) (Fig. 1) is located at the eastern shore side of the Rock of Gibraltar in the southernmost part of the Iberian Peninsula. Several stratigraphic levels with archaeological evidence of Neanderthals and Modern Humans have been identified: Levels I and II belong to the Holocene, with significant Phoenician and Carthaginian artefacts. Level III is Upper Palaeolithic, and Level IV is Mousterian, which is associated with Neanderthals in Western Europe^51^,^52^.

Vanguard Cave (36° 07´N, 05°20´W) (Fig. 1) is located on the eastern side of the Rock of Gibraltar, adjacent to the Gorham’s Cave. The site contains a sequence of about 17 metres of sandy sediments with animal and plant fossils, and Mousterian lithic tools, also indicating a Neanderthal occupation. A number of well-stratified occupation beds, containing hearths and vertebrate and invertebrate fossils, as well as pollen and charcoal remains, are being studied. Preliminary data from OSL ages suggest an age from ∼120 to ∼75 ky^26^.

El Pirulejo cave (37°, 26´N, 04° 11’W and 580 m asl) (Fig. 1), is located in the town of Priego de Córdoba in the southern part of Spain and lies within a travertine formation close to the northern edge of the Betic Range and the Guadalquivir Basin. This site was the refuge of Palaeolithic populations from ∼17 to 14 cal ky BP. The site is also known by its prominent Bronze Age occupation^45^.

### Used techniques

Forty-four sedimentary samples from the selected archaeological sites, as well as three samples from ashes obtained by experimental fires were analysed. Analyses of thirty nine trace elements (Li, Rb, Cs, Be, Sr, Ba, Sc, V, Cr, Co, Ni, Cu, Zn, Ga, Y, Nb, Ta, Zr, Hf, Mo, Sn, Tl, Pb, U, Th, La, Ce, Pr, Nd, Sm, Eu, Gd, Tb, Dy, Ho, Er, Tm, Yb and Lu) were carried out by ICP-MS (Perkin- Elmer Sciex, Elan 5000) at the Centro de Instrumentación Científica of the University of Granada. Rh and Re were used as internal standards. The relative error of this device was ±2% and ±5% connected with element concentration of 50 ppm and 5 ppm respectively.

Zn isotopic ratios were measured on a Nu Plasma MC-ICP-MS at the “Université Libre de Bruxelles”, Belgium. The analyses were performed in the wet plasma mode. Mass discrimination effects were corrected by simultaneously external normalization (Cu-doping method) and sample-standard bracketing. All Zn isotopic results are reported in the conventional δ^66^Zn notation (δ^66^Zn = ([(^66^Zn/^64^Zn)_sample_/[(^66^Zn/^64^Zn)_standard_]–1) × 1000) relative to the JMC 3-0749L Zn and NIST SRM 976 Cu reference standards. The mean value obtained from the JMC 3-0749L Zn standard solution relative to our in-house Zn standard is + 0.11 ± 0.03‰ (2SD) (n = 17). During data collection, repeated analyses of our in-house Zn standard and IRMM Zn 3702 standard solutions gave the mean values of 0.00 ± 0.03‰ (2SD) (n = 81) and + 0.45 ± 0.02‰ (2SD) (n = 2), respectively.

Heavy metals are presently considered to be those elements with an atomic weight greater than that of Fe (>55,85 g/mol). Heavy metal pollution means that the concentration of the element is higher than the established threshold, or natural geochemical background in a given area^53^.

In order to assess the possible polluted levels, the recommendations from the Soil Cleanup Criteria^16^, and the parameter Enrichment Factor (EF), also called Anthropic Factor^54^,^55^,^56^ were taken into account. EF reflects the degree of relative contamination of an element related to its geochemical baseline in the area. It is expressed as: **EF** = Ce/Be, where Ce is the measured element concentration in soil or sediment and Be is the geochemical baseline in each site of study. The geochemical baseline was defined on the basis of the data reported for the Southern Iberia (Supplementary Table S1)^57^ and those from the Northern Iberia^58^ taken from different soils and geological outcroups along the entire regions.

Experimental fires with wood from the arboreal *Quercus faginea*, *Quercus ilex* and *Pinus pinea* species were performed. These taxa are representative of the studied archaeological sites, according to pollen and charcoal analyses^59^. The collected tree samples come from the Natural Park “Sierra de Grazalema”, from trees which grew on limestone soils and far away from sources of pollution. Wood remains were rinsed with distilled water in order to remove surface pollutants. Ashes were obtained from 8 g of each specimen at 650 ± 10 °C during two hours, with a heating gradient of 5 °C min^−1^ by means of an electric oven (Carbolite). In addition, the ashes were geochemically analysed to find out their hypothetical contribution to the sedimentary sample collection.

## Additional Information

**How to cite this article**: Monge, G. *et al.* Earliest evidence of pollution by heavy metals in archaeological sites. *Sci. Rep.* **5**, 14252; doi: 10.1038/srep14252 (2015).

## Supplementary Material

Supplementary Information
