# Supplementary material for: Earliest evidence of pollution by heavy metals in archaeological sites
Source: Sci Rep. 2015 Sep 21;5:14252. doi: 10.1038/srep14252 (PMC4585679; doi:10.1038/srep14252)
Supplement: Supplementary Information [file srep14252-s1.doc]

**Supplementary Information**

**Earliest evidence of pollution by heavy metals in archaeological sites**

Guadalupe Monge, Francisco J. Jimenez-Espejo, Antonio García-Alix, Francisca Martínez-Ruiz, Nadine Mattielli, Clive Finlayson, Naohiko Ohkouchi, Miguel Cortés Sánchez, Jose María Bermúdez de Castro*,* Ruth Blasco, Jordi Rosell, José Carrión, Joaquín Rodríguez-Vidal, Geraldine Finlayson

**Table S1.** Selected trace element content and Enrichment Factor (EF) values of the studied archaeological sites, experimental tree ashes and compiled geochemical base lines. Bold number and grey dashed area indicate values with EF≥ 2.0.

| Site Locat. | Sample id. | Level/Grid/Depth | Culture | Cr (ppm) | EF(Cr) | Ni (ppm) | EF (Ni) | Cu (ppm) | EF(Cu) | Zn (ppm) | EF(Zn) | Pb (ppm) | EF(Pb) |
| --- | --- | --- | --- | --- | --- | --- | --- | --- | --- | --- | --- | --- | --- |
| Gran Dolina | TD 9 GQ 10 | 9 / 520 |  | 65 | 1,2 | 17 | 0,7 | 138 | **2,8** | 247 | **3,7** | 22 | 0,4 |
| Gran Dolina | TD 9 GQ 11 | 9 / 560 |  | 48 | 0,9 | 46 | 1,8 | 292 | **5,8** | 614 | **9,2** | 16 | 0,3 |
| Gran Dolina | TD 6-GQ 14 | 2 | Low. Palaeol. | 59 | 1,1 | 41 | 1,6 | 91 | 1,8 | 299 | **4,5** | 22 | 0,4 |
| Gran Dolina | TD 6-GQ 15 | 2 | Low. Palaeol. | 40 | 0,8 | 28 | 1,1 | 82 | 1,6 | 304 | **4,5** | 15 | 0,3 |
|  |  |  |  |  |  |  |  |  |  |  |  |  |  |
| Gorham´s | GOR-7 | I / AA9 /205 | Neolithic | 66 | 0,9 | 49 | 1,7 | 81 | **3,4** | 342 | **6,1** | 22 | 0,9 |
| Gorham´s | GOR-8 | II / AA9 /212 | Up. Palaeol. | 70 | 1,0 | 49 | 1,7 | 91 | **3,8** | 354 | **6,3** | 30 | 1,3 |
| Gorham´s | GOR-13 | II / AA9 / 232 | Up. Palaeol. | 73 | 1,0 | 48 | 1,6 | 68 | **2,8** | 293 | **5,2** | 15 | 0,6 |
| Gorham´s | GOR-5 | IIIA / AA4 / 235 | Up. Palaeol. | 81 | 1,2 | 59 | **2,0** | 127 | **5,3** | 530 | **9,5** | 26 | 1,1 |
| Gorham´s | GOR-10 | IIIA / AA9 / 235 | Up. Palaeol. | 72 | 1,0 | 44 | 1,5 | 60 | **2,5** | 248 | **4,4** | 15 | 0,6 |
| Gorham´s | GOR-11 | IIIB / AA9 / 265 | Up. Palaeol. | 74 | 1,1 | 52 | 1,8 | 72 | **3,0** | 311 | **5,5** | 18 | 0,8 |
| Gorham´s | GOR-14 | IIIB / AA6 /265 | Up. Palaeol. | 89 | 1,3 | 59 | **2,0** | 112 | **4,7** | 487 | **8,7** | 21 | 0,9 |
| Gorham´s | GOR-12 | IV / AA9 /305 | Mid. Palaeol. | 112 | 1,6 | 202 | **7,0** | 74 | **3,1** | 687 | **12,3** | 18 | 0,8 |
| Gorham´s | GOR-1 | IV / AA5 / 270 | Mid. Palaeol. | 108 | 1,5 | 75 | **2,6** | 134 | **5,6** | 1161 | **20,7** | 26 | 1,1 |
| Gorham´s | GOR-2 | IV / AA2 | Mid. Palaeol. | 98 | 1,4 | 494 | **17,0** | 1593 | **66,4** | 4158 | **74,3** | 13 | 0,5 |
|  |  |  |  |  |  |  |  |  |  |  |  |  |  |
| Vanguard | V-1 | 4 / K61 / 80 | Mid. Palaeol. | 60 | 0,9 | 30 | 1,0 | 9 | 0,4 | 37 | 0,7 | 4 | 0,2 |
| Vanguard | V-2 | 5 / K61 / 84 | Mid. Palaeol. | 79 | 1,1 | 44 | 1,5 | 12 | 0,5 | 69 | 1,2 | 6 | 0,2 |
| Vanguard | V-3 | 5 / K61 / 105 | Mid. Palaeol. | 128 | 1,8 | 61 | **2,1** | 14 | 0,6 | 61 | 1,1 | 8 | 0,3 |
| Vanguard | V-4 | 5 / K61 / 143 | Mid. Palaeol. | 106 | 1,5 | 52 | 1,8 | 14 | 0,6 | 57 | 1,0 | 7 | 0,3 |
| Vanguard | V-7 | 9 / J58 / 208 | Mid. Palaeol. | 112 | 1,6 | 49 | 1,7 | 22 | 0,9 | 90 | 1,6 | 7 | 0,3 |
| Vanguard | V-21 | 9 / M58 / 210 | Mid. Palaeol. | 146 | **2,1** | 82 | **2,8** | 45 | 1,9 | 177 | **3,2** | 11 | 0,5 |
| Site Locat. | Sample id. | Level/Grid/Depth | Culture | Cr (ppm) | EF(Cr) | Ni (ppm) | EF (Ni) | Cu (ppm) | EF(Cu) | Zn (ppm) | EF(Zn) | Pb (ppm) | EF(Pb) |
| Vanguard | V-9 | 9 / K58 / 211 | Mid. Palaeol. | 165 | **2,4** | 66 | **2,3** | 120 | **5,0** | 229 | **4,1** | 11 | 0,5 |
| Vanguard | V-10 | 9 / K58 / 223 | Mid. Palaeol. | 143 | **2,0** | 58 | **2,0** | 53 | **2,2** | 207 | **3,7** | 9 | 0,4 |
| Vanguard | V-12 | 10 / J58 / 235 | Mid. Palaeol. | 83 | 1,2 | 29 | 1,0 | 7 | 0,3 | 16 | 0,3 | 3 | 0,1 |
| Vanguard | V-16 | 10 / J58 / 255 | Mid. Palaeol. | 151 | **2,2** | 66 | **2,3** | 18 | 0,7 | 50 | 0,9 | 8 | 0,3 |
| Vanguard | V-17 | 12 / J58 / 272 | Mid. Palaeol. | 175 | **2,5** | 47 | 1,6 | 8 | 0,3 | 23 | 0,4 | 3 | 0,1 |
| Vanguard | V-22 | 12 / M58 / 280 | Mid. Palaeol. | 133 | 1,9 | 40 | 1,4 | 8 | 0,3 | 16 | 0,3 | 4 | 0,2 |
| Vanguard | V-18 | 13 / J58 / 282 | Mid. Palaeol. | 161 | **2,3** | 43 | 1,5 | 7 | 0,3 | 16 | 0,3 | 3 | 0,1 |
| Vanguard | V-19 | 14 / J58 / 300 | Mid. Palaeol. | 130 | 1,9 | 63 | **2,2** | 20 | 0,8 | 58 | 1,0 | 8 | 0,3 |
| Vanguard | V-23 | 15 / M58 / 309 | Mid. Palaeol. | 134 | 1,9 | 46 | 1,6 | 8 | 0,3 | 18 | 0,3 | 4 | 0,2 |
| Vanguard | V-20 | 15 / J58 / 311 | Mid. Palaeol. | 140 | **2,0** | 56 | 1,9 | 15 | 0,6 | 44 | 0,8 | 6 | 0,3 |
|  |  |  |  |  |  |  |  |  |  |  |  |  |  |
| El Pirulejo | P-80 | I / T4 / 80 | Bronze Age | 40 | 0,6 | 49 | 1,7 | 23 | 0,9 | 57 | 1,0 | 60 | **2,5** |
| El Pirulejo | P-85 | I / T4 / 85 | Bronze Age | 18 | 0,3 | 40 | 1,4 | 19 | 0,8 | 48 | 0,9 | 44 | 1,8 |
| El Pirulejo | P-100 | I / T4 / 100 | Bronze Age | 45 | 0,6 | 48 | 1,6 | 21 | 0,9 | 50 | 0,9 | 77 | **3,2** |
| El Pirulejo | P-110 | I / T4 / 110 | Bronze Age | 18 | 0,3 | 39 | 1,4 | 15 | 0,6 | 38 | 0,7 | 86 | **3,6** |
| El Pirulejo | P-132 | II / T4 / 132 | Last Magalen. | 21 | 0,3 | 40 | 1,4 | 14 | 0,6 | 36 | 0,6 | 54 | **2,3** |
| El Pirulejo | P-140 | III / T4 / 140 | Up. Magalen. | 170 | **2,4** | 96 | 3,3 | 17 | 0,7 | 31 | 0,6 | 78 | **3,3** |
| El Pirulejo | P-165 | III / T4 / 165 | Up. Magalen. | 18 | 0,3 | 38 | 1,3 | 11 | 0,5 | 41 | 0,7 | 38 | 1,6 |
| El Pirulejo | P-180 | IV / T4 / 180 | Mid. Magalen. | 140 | **2,0** | 125 | **4,3** | 22 | 0,9 | 41 | 0,7 | 56 | **2,4** |
| El Pirulejo | P-200 | IV / T4 / 200 | Mid. Magalen. | 27 | 0,4 | 38 | 1,3 | 15 | 0,6 | 34 | 0,6 | 7 | 0,3 |
| El Pirulejo | P-210 | IV / T4 / 210 | Mid. Magalen. | 22 | 0,3 | 41 | 1,4 | 15 | 0,6 | 43 | 0,8 | 12 | 0,5 |
| El Pirulejo | P-225 | IV / T4 / 225 | Mid. Magalen. | 32 | 0,5 | 55 | 1,9 | 21 | 0,9 | 37 | 0,7 | 8 | 0,3 |
| El Pirulejo | P-250 | V / T4 / 250 | Solutrean | 20 | 0,3 | 37 | 1,3 | 13 | 0,6 | 21 | 0,4 | 7 | 0,3 |
| El Pirulejo | P-270 | V / T4 / 270 | Solutrean | 30 | 0,4 | 38 | 1,3 | 12 | 0,5 | 24 | 0,4 | 6 | 0,2 |
| El Pirulejo | P-280 | VI / T4 / 280 | Up. Palaeol. | 14 | 0,2 | 39 | 1,4 | 6 | 0,3 | 12 | 0,2 | 3 | 0,1 |
|  |  |  |  |  |  |  |  |  |  |  |  |  |  |
| Site Locat. | Sample id. | Level/Grid/Depth | Culture | Cr (ppm) | EF(Cr) | Ni (ppm) | EF (Ni) | Cu (ppm) | EF(Cu) | Zn (ppm) | EF(Zn) | Pb (ppm) | EF(Pb) |
|  |  |  |  |  |  |  |  |  |  |  |  |  |  |
| *Quercus ilex*/Ashes | Q. i. |  |  | 23 |  | 39 |  | 133 |  | 492 |  | 13 |  |
| *Pinus Pinea*/Ashes | P. p. |  |  | 35 |  | 48 |  | 127 |  | 717 |  | 17 |  |
| *Quercus faginea* /Ashes | Q. f. |  |  | 28 |  | 54 |  | 85 |  | 281 |  | 26 |  |
| Geochem. Baseline South Iberia |  |  |  | 70 |  | 29 |  | 24 |  | 56 |  | 24 |  |
| Geochem. Baseline Central Iberia |  |  |  | 53 |  | 26 |  | 50 |  | 67 |  | 51 |  |
|  | |  |  |  |  |  |  |  |  |  |  |  |  |
|  | |  |  |  |  |  |  |  |  |  |  |  |  |

**Table S2.** δ^66^Zn and other Zn isotopic values of samples GOR-2 (Neanderthal´s hearth) and GOR-12 from Gorham´s Cave.

| Site location | Sample id. | δ^68^Zn | 2SD | δ^66^Zn | 2SD | Avrg δ^68^Zn | 2SD | Avrg δ66 Zn | 2SD | Number of replicates |
| --- | --- | --- | --- | --- | --- | --- | --- | --- | --- | --- |
|  | GOR 2 a1 | +1,78 | 0,06 | +0,91 | 0,02 |  |  |  |  |  |
|  |  |  |  |  |  |  |  |  |  |  |
| Gorham´s | GOR 2 a2 | +1,81 | 0,06 | +0,91 | 0,02 | **+1,79** | 0,05 | **+0,91** | 0,02 | n=2 |
|  |  |  |  |  |  |  |  |  |  |  |
|  |  |  |  |  |  |  |  |  |  |  |
|  |  |  |  |  |  |  |  |  |  |  |
|  | GOR 2 b1 | +1,73 | 0,08 | +0,89 | 0,03 | **+1,77** | 0,12 | **+0,90** | 0,03 | n=2 |
|  |  |  |  |  |  |  |  |  |  |  |
|  | GOR 2 b2 | +1,81 | 0,05 | +0,91 | 0,02 |  |  |  |  |  |
|  |  |  |  |  |  |  |  |  |  |  |
|  | GOR 12 1 | +1,26 | 0,05 | +0,63 | 0,01 |  |  |  |  |  |
|  |  |  |  |  |  |  |  |  |  |  |
|  | GOR 12 2 | +1,30 | 0,05 | +0,64 | 0,02 | **+1,28** | 0,04 | **+0,63** | 0,01 | n=3 |
|  |  |  |  |  |  |  |  |  |  |  |
|  | GOR 12 3 | +1,28 | 0,07 | +0,63 | 0,02 |  |  |  |  |  |
